# An immunoinformatics study: designing multivalent T-cell epitope vaccine against canine circovirus

**DOI:** 10.1186/s43141-021-00220-4

**Published:** 2021-08-18

**Authors:** Pankaj Jain, Amit Joshi, Nahid Akhtar, Sunil Krishnan, Vikas Kaushik

**Affiliations:** Domain of Bioinformatics, School of Bioengineering and Biosciences, Lovely Professional University, Phagwara, Punjab India

**Keywords:** Canine circovirus, Epitope, Vaccine designing, Molecular docking, Molecular dynamic simulations, Dog leukocyte antigen

## Abstract

**Background:**

*Canine circovirus* is a deadly pathogen of dogs and causes vasculitis and hemorrhagic enteritis. It causes lethal gastroenteritis in pigs, fox, and dogs. Canine circovirus genome contains two main (and opposite) transcription units which encode two open reading frames (ORFs), a replicase-associated protein (Rep) and the capsid (Cap) protein. The replicase protein and capsid protein consist of 303 amino acids and 270 amino acids respectively. Several immuno-informatics methods such as epitope screening, molecular docking, and molecular-dynamics simulations were used to craft peptide-based vaccine construct against canine circovirus.

**Results:**

The vaccine construct was designed by joining the selected epitopes with adjuvants by suitable linker. The cloning and expression of the vaccine construct was also performed using in silico methods. Screening of epitopes was conducted by NetMHC server that uses ANN (Artificial neural networking) algorithm. These methods are fast and cost-effective for screening epitopes that can interact with dog leukocyte antigens (DLA) and initiate an immune response. Overall, 5 epitopes, YQHLPPFRF, YIRAKWINW, ALYRRLTLI, HLQGFVNLK, and GTMNFVARR, were selected and used to design a vaccine construct. The molecular docking and molecular dynamics simulation studies show that these epitopes can bind with DLA molecules with stability. The codon adaptation and in silico cloning studies show that the vaccine can be expressed by *Escherichia coli* K12 strain.

**Conclusion:**

The results suggest that the vaccine construct can be useful in preventing the dogs from canine circovirus infections. However, the results need further validation by performing other in vitro and in vivo experiments.

## Background

Circoviruses are unenveloped, spherical viruses with a diameter of about 20 nm and a circular, single-stranded DNA genome of about 2 kb belonging to the Circoviridae family [1]. *Canine circovirus* (CanineCV) [2] or *dog circovirus* (Dog CV) belong to the genus *Circovirus*. They are the smallest known autonomously replicating, capsid encoding animal pathogens. Their genome contains two main (and opposite) transcription units which encode two open reading frames (ORFs), a replicase-associated protein (Rep) and the capsid (Cap) protein [3]. This virus replicates its genomes using a circular, ds replicative form (RF) DNA intermediate which is produced using host cell DNA polymerases during the S phase of cell division. The RF serves as a template for the generation of viral ssDNA, probably using the rolling circle replication (RCR) mechanism. Their genome consists of two coding and two noncoding parts. For unique viral replicase and capsid proteins, there are only two open reading frames (ORFs). Viral replicase protein and capsid protein consist of 303 amino acids and 270 amino acids respectively. A series of 30 arginine amino acids from the amino terminus is used in the gene coding for the capsid. For DNA binding, this particular stretch is hypothesized to be important [4, 5]. Canine circovirus has been described in cases of dogs with vasculitis and/or hemorrhagic enteritis in the USA and Italy [6, 7]. Regardless of the prevalence of circovirus infection, the pathogenic role of canine circovirus in single or polymicrobial infections is undetermined as well as the prevalence of this virus in other wild carnivores [8]. A closely related circovirus was detected in foxes with meningoencephalitis in the UK. The pathogenesis of canine circovirus infection is incompletely understood [9]. Canine circovirus has only been detected in the USA [7, 10], Italy [6], Germany (GenBank accession number: KF887949), and China (GenBank accession number: KT946839) [11]. Song et al. isolated Porcine circovirus type 2 (PCV2) in raccoon dogs (*Mangut*) in China which caused failure in reproduction of raccoon dogs and concluded the route of transmission of PCV2 from pigs to raccoon dogs [12]. Kotsias et al. isolated canine circovirus along with *canine parvovirus* (CPV) in samples from an outbreak of fatal gastroenteritis in dogs in Argentina (South America) and showed after phylogenetic analysis that UBA-Baires strain is closely related to European strains than to viruses detected in North America or Asia [3]. As canine circovirus can be fatal to the dog population, it is imperative to look for novel ways to combat their infections. One such strategy can be developing vaccine candidates that can prevent dogs from canine circovirus infections by generating a robust immune response. Hence, in this study, a multi epitope vaccine construct has been designed against canine circovirus by targeting their replicase and capsid proteins via in silico approach. As, these proteins are highly conserved, are involved in viral pathogenesis, and can help to generate immune response, they can be a good target for identifying epitopes that could be used to design a vaccine construct which can help protect the dog population from potentially lethal canine circovirus infections [13–15]. Such in silico studies have been previously performed to design vaccine construct against dengue, *Candida auris*, human cytomegalovirus, SARS-CoV-2, Lassa virus, human papillomavirus, cervical cancer, and hepatitis C virus [16–26]. In our study, various immunoinformatic tools were used to design a potential vaccine construct against canine circovirus. First highly antigenic epitopes were selected and their interaction with dog leukocyte antigen (DLA) molecules was analyzed by molecular docking and molecular dynamics simulation studies. Then antigenic epitopes were linked with RS09 and flagellin adjuvants along with PADRE sequence by GGS linkers to construct a vaccine candidate. The physiochemical properties, antigenicity, allergenic potential, and secondary and tertiary structure of the designed vaccine construct were also predicted using different webservers. Finally, the ability of the vaccine construct to be cloned and expressed was also analyzed by in silico cloning method. This vaccine construct could be beneficial in protecting the dogs from canine circovirus infections.

## Methods

### Proteomic data retrieval

ViPR database was used as it is based on IEDB for database assessment that assisted us in gathering information regarding viral proteome. The viral replicase and capsid proteins structural sequences of canine circovirus were retrieved from the NCBI-Proteomic database with accession ID’s: QFU80922.1 replicase [*Canine circovirus*] and QBQ20241.1 capsid Protein [*Canine circovirus*] (Table 1).

**Table 1:** Retrieved FASTA sequences

>QFU80922.1 replicase [*Canine circovirus*] MAQAQVDQRGRDSRRGNPVRRWCFTINNPTPEEEEAVKNLAPDAKYLICGREVGENGTPHLQGFVNLKKTTRMGALKARLGGRGHFEPARGDDCSNKDYCSKGGDILIESGEVSRQGKRNDLHDAVEKLRETKSLAAVAAAYPETYVKFSRGLRELLLISPEMTTPRNWKTEVEVLCGPPGCGKSRYCMETAPDAYWKPRGKWWDGYDGHQDVILDDFYGWLPFDDMLRLCDRYPLRVETKGGTMNFVARRVFITSNRLPHEWYSDEIGNKDALYRRLTLIKVWDGGNFIPVPHFMFPHMYNY	
>QBQ20241.1 capsid Protein [*Canine circovirus*] MRVRRHARASRRSYRTRPLNRYRRRRQNRFKLFHLRLRRTLTADWPTAPVKPTNDPQTETPLLWNFDHLSFKLTDFLQTSHGTGDYQHLPPFRFFKFKKVYIRAKWINWPRTLMENVLGRTALDLDGEDQGRGNAQRSHLDPGCVPGRLEPPKDPNKAPFIYDPLQDRSSSRSFNMASGFKRGLTPKPMFTQDITSPSATAPWLTRGTPWVSVIQGANMVWNGLSISLRQMKDMRPTTPDTSTSQIPQVQYDISAYIAFKEFDYETGRQL	

### Epitopes selection and structure prediction

The NetMHCpan-4.1 server predicts peptides/epitopes from viral proteomic determinants by using artificial neural networks to varied MHC molecule with a familiar sequence (ANNs) [27]. To predict the epitopes, dog MHC molecules DLA-8803401, DLA-8850101, and DLA-8850801 were selected. The predicted epitopes were selected on the basis of bind level, i.e., if they are strong binder or weak binder. The weak and strong binders were determined by using the default parameters of the webserver. By default, a peptide will be predicted as strong binder if its percentage rank is below 0.5% and a peptide will be predicted as weak binder if its percentage rank is above 0.5% but below 2% rank [27]. VaxiJen was also used in this study to determine the antigenic potential of the selected epitope [28]. Then, selected epitopes structural prediction was conducted by using PEP-FOLD, a de novo approach aimed at predicting peptide structures from amino acid sequences. This approach couples the deep learning algorithm and a coarse-grained force field based on amino acids to determine the conformations of consecutive amino acid residues as per their physiochemical relationships in secondary and tertiary folding [29].

### Molecular docking and simulation at epitope-DLA level

For molecular docking between DLA proteins and epitopes, PatchDock webserver [30] and Autodock Vina [31] were used. The structures for DLA allelic sets were retrieved from the RCSB-PDB database. DLA alleles DLA-8803401 and DLA-8850801 were considered for performing the molecular docking and simulation analysis. The PDB ID of DLA-8803401 and DLA-8850801 are “7CJQ” and “5F1I” respectively. For molecular docking and simulation studies, the PDB file of the epitopes and DLA were used. PatchDock assists the user to determine atomic contact energies, and Autodock vina assists the user to determine binding energies for perfectly docked complexes. Furthermore, docking of the epitopes with DLA molecules was also performed using ClusPro 2.0 webserver [32]. ClusPro is a protein-protein docking server which results in 10 models of the docking complex by defining the centers of highly populated clusters of low energy docked structures [32], while for stability analysis of docked complexes molecular simulation was performed by deploying Gromacs [33].

### Full-fledged in silico vaccine construction

The final vaccine construct was designed by linking the selected antigenic epitopes by GGS linkers to RS09 and *Salmonella dublin flagellin* protein as adjuvants. Pan HLA DR-binding epitope (PADRE) sequence was also added to vaccine construct for providing stability to the vaccine construct. The final vaccine construct was then subjected to stability analysis and physio-chemical characteristics determination by deploying the “Expasy ProtParam” [34]. The solubility of the vaccine was evaluated by the Solpro web server [35]. The Solpro web server predicts the solubility of proteins with 74% accuracy after expression in *Escherichia coli* [35]*.*

### Vaccine construct prediction and affirmation

The secondary structure of vaccine was determined by using PSIPRED webserver [36]. This web server helps in the prediction of beta-sheets, alpha helices, and coils in proteins by using feed-forward neural networks [36]. The tertiary structure of the vaccine was determined by using the I-TASSER server which uses iterative template-based fragments assembly simulations and multiple threading approaches to predict the 3D model of proteins [37] and uses iterative template-based fragments assembly simulations and multiple threading approaches to predict the 3D model of proteins. Finally, the tertiary structure of the modeled vaccine was validated by the Procheck web server [38].

### Codon adaptation and vaccine in silico cloning

For efficient cloning and expression of the vaccine in expression vectors, codon optimization is imperative. JCAT web server was used for codon optimization of vaccine for expression in *E. coli* K12 strain [39]. The “SnapGene” restriction cloning module was used for in silico cloning of vaccine.

## Results

### Proteome structure and sequence

We retrieved FASTA sequences of protein from the NCBI database as summarized in Table 1.

### Predicted epitopes and DLA structure

NetMHCpan 4.1 server was deployed to find out interacting epitopes; lowest values were preferred while selecting epitopes based on prediction scores. For viral replicase 294 epitopes interacting with each DLA, the allele was identified, while for viral capsid protein 261 epitopes interacting with each DLA allele were identified. Out of these 555 epitopes, the best 20 were considered based on epitope prediction scores. VaxiJen server was also used for determining antigenicity with the threshold value of 0.7. Five epitopes were selected out of 20 epitopes based on their antigenicity (Table 2). Structures were constructed by deploying the PEP-FOLD server, which follows de novo criteria for predicting perfect conformation for epitopes. The structures for DLA allelic sets were retrieved from the RCSB-PDB database; two major DLA alleles were considered during investigation DLA-8803401 and DLA-8850801 with PDB IDs “7CJQ” and “5F1I” respectively. Both the structures of DLA alleles were crystal structure and can be easily accessed by their unique PDB IDs.

**Table 2:** Epitopes selection based on Epitope prediction score and antigenicity score

Protein	DLA type	Epitope	Position of epitopes on the protein sequence	Epitope prediction score	VaxiJen antigenicity score (threshold value, 0.7)	Select/reject
Viral replicase	DLA-8803401	FMFPHMYNY	295–303	0.8249350	0.5493	Reject
		ALYRRLTLI	273–281	0.5142250	0.7123	Select
		RVFITSNRL	251–259	0.4589590	0.0434	Reject
		KVWDGGNFI	282–290	0.3829500	− 0.4171	Reject
Capsid protein	DLA-8803401	YQHLPPFRF	86–94	0.4774550	0.9208	Select
		YIRAKWINW	101–109	0.3914190	2.2087	Select
Viral replicase	DLA-8850101	FMFPHMYNY	295–303	0.6891070	0.5493	Reject
		RVFITSNRL	251–259	0.5503590	0.0434	Reject
		KVWDGGNFI	282–290	0.5206210	− 0.4171	Reject
Capsid protein	DLA-8850101	SVIQGANMV	212–220	0.4310240	0.5916	Reject
Viral replicase	DLA-8850801	FMFPHMYNY	295–303	0.7916770	0.5493	Reject
		AAYPETYVK	140–148	0.7143770	0.3805	Reject
		GTMNFVARR	243–251	0.6422870	0.7786	Select
		HLQGFVNLK	60–68	0.5264080	0.8062	Select
		KVWDGGNFI	282–290	0.4437560	− 0.4171	Reject
Capsid protein	DLA-8850801	FIYDPLQDR	160–168	0.6825130	− 0.0399	Reject
		HLPPFRFFK	88–96	0.6528390	− 0.2234	Reject
		TLMENVLGR	112–120	0.6319340	− 0.7453	Reject
		KLFHLRLRR	31–39	0.6040340	0.6741	Reject
		VQYDISAYI	249–257	0.5071050	0.1127	Reject

### Molecular docking and simulation

**Fig. 1:**
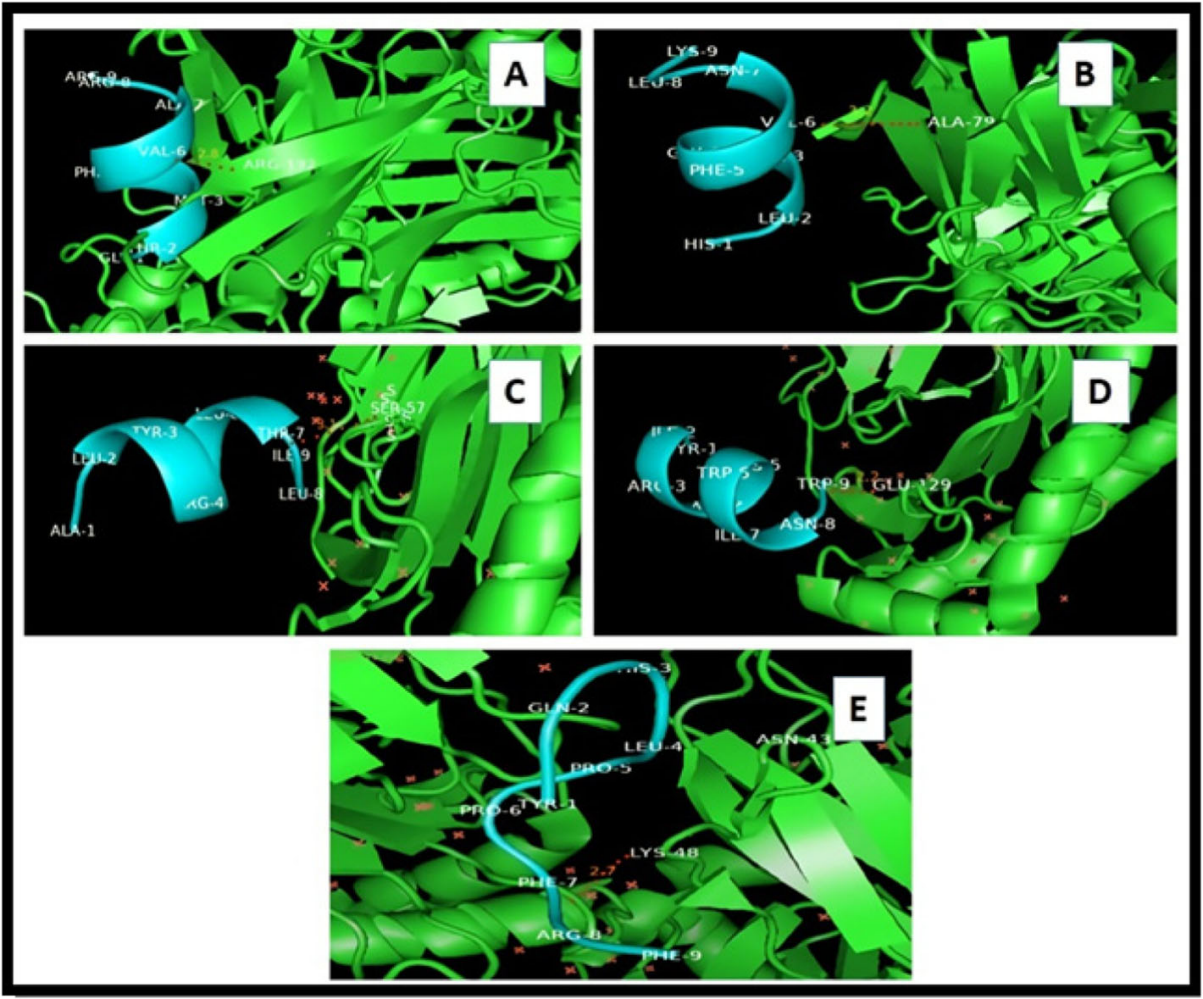
Docked complexes: **A** 5F1I-GTMNFVARR (2.8ÅHydrogen bond Arg192 to Val6). **B** 5F1I-HLQGFVNLK (3.2Å Hydrogen bond Ala79 to Val6). **C** 7CJQ-ALYRRLTLI (3.1Å Hydrogen bond Ser57 to Thr7). **D** 7CJQ-YIRAKWINW (2.2Å Hydrogen bond Glu129 to Trp9). **E** 7CJQ-YQHLPPFRF (2.7Å Hydrogen bond Lys48 to Phe7)

Docking studies were conducted to determine the efficiency of binding or interaction between DLA alleles and respective epitopes. Most negative ACE (atomic contact energy) values and binding energies indicate perfect interaction between the selected epitopes and DLA alleles (Table 3). The 5 best possible docked complexes were showed perfect hydrogen bond interactions during visualization analysis by deploying PyMol visualizing tool. All the H-bond interactions are shown in Fig. 1. The bonding criteria for 5 selected complexes were found, as follows: 5F1I-GTMNFVARR (2.8Å Hydrogen bond Arg192 to Val6), 5F1I-HLQGFVNLK (3.2Å Hydrogen bond Ala79 to Val6), 7CJQ-ALYRRLTLI (3.1Å Hydrogen bond Ser57 to Thr7), 7CJQ-YIRAKWINW (2.2Å Hydrogen bond Glu129 to Trp9), and 7CJQ-YQHLPPFRF (2.7Å Hydrogen bond Lys48 to Phe7).

**Table 3:** DLA-epitope complex docking analysis: ACE values and binding energy values for best possible complexes

DLA-epitope docked complex	Atomic contact energy	Binding energy (Kcal/mol)	ClusPro lowest energy
7CJQ-YQHLPPFRF	− 147.66	− 7.5	− 688.7
7CJQ-YIRAKWINW	− 55.05	− 6.8	− 745.4
7CJQ-ALYRRLTLI	− 107.87	− 7.8	− 781.7
5F1I-HLQGFVNLK	− 168.42	− 8.8	− 741.4
5F1I-GTMNFVARR	− 64.97	− 7.1	− 671.7

**Fig. 2:**
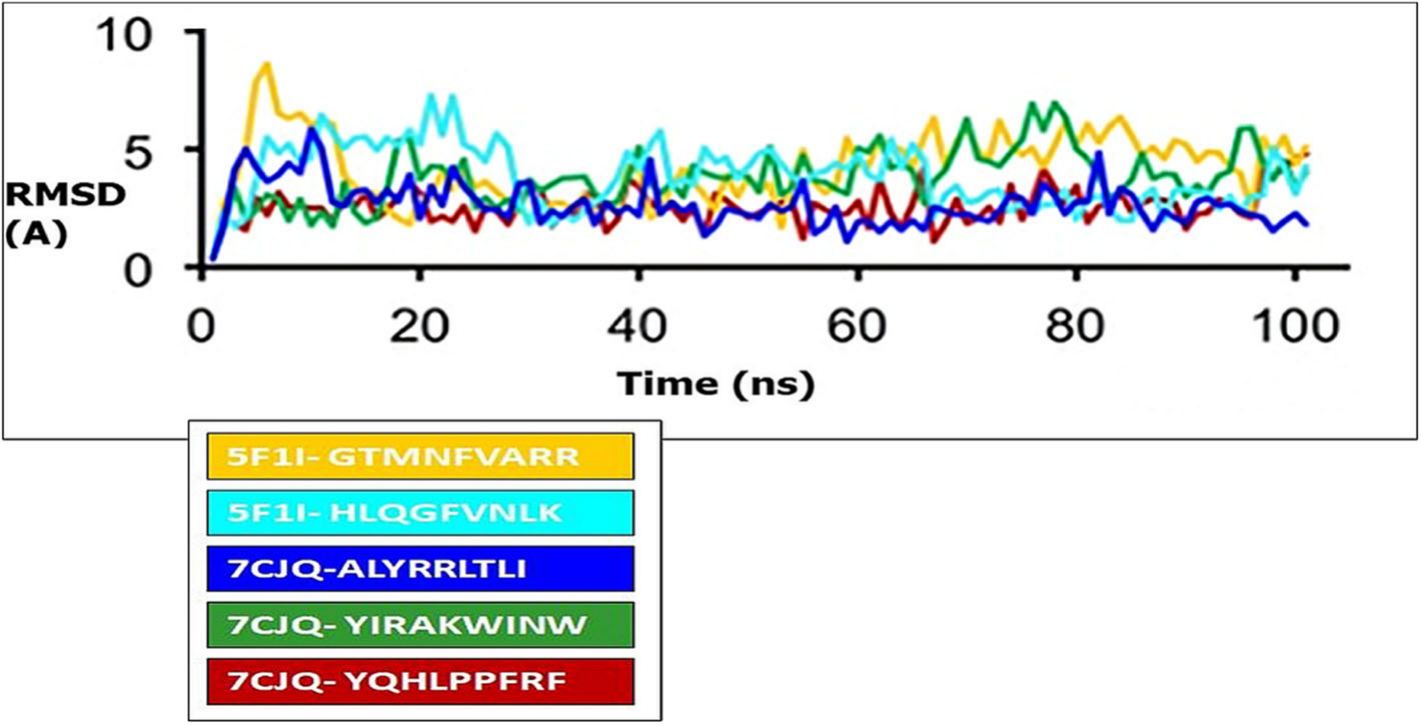
RMSD plot for 5 selected epitopes-DLA complexes

With the help of Gromacs tool, molecular dynamics (MD) simulations were performed using OPLS-AA field. OPLS-AA counts all atoms of docked complexes. The complex was stabilized towards the edges in a cubical box having a distance of 1.5 nm applying clear TIP4P water model. Charges were neutralized by adding Na+ ions in this biomolecular simulation. Steepest descent technique of 200,000 steps with 0.001 nm initial step-size was employed for energy minimization. For MD simulations, leap-frog algorithm to assimilate Newton’s equations was used. Using LINCS (LINear Constraint Solver) algorithm and periodic boundary conditions (PBC) in all directions, the bond lengths were maintained. Cut-off of 0.9 nm distance and Particle-Mesh Ewald (PME) methods were used for the short-range interactions and for long-range interactions respectively. The temperature (300 K) and pressure (1 bar) were regulated by V-rescale thermostat and Parrinello-Rahman barostat respectively. Molecular dynamics used in the isobaric-isothermal ensemble calculations without position restraint during 100 ns. Root mean square deviation (RMSD) plots indicate the selectable range of 0 to 8 Å for all considered epitopes in Fig. 2.

### Full-fledged vaccine construction

The epitopes that were predicted as highly antigenic and could bind with the DLA molecules were selected for the vaccine design. RS09 and N and C terminal of *Salmonella Dublin* flagellin protein (UNIPROT ID: Q06971) were used as adjuvants. PADRE sequence was incorporated in the vaccine to develop stability and linked to each other by the GGS linker sequence. The final vaccine sequence is summarized in Table 4. Then, the physiochemical properties like stability, number of amino acids, molecular weight, isoelectric point, aliphatic index, number of positively and negatively charged amino acids, and various other properties and also the solubility of the vaccine were evaluated by the Solpro as summarized in Table 5. The vaccine construct was predicted as antigenic, non-allergic, stable, and soluble.

**Table 4:** The full-fledged vaccine sequence

**Final vaccine construct**	
MAQVINTNSLSLLTQNNLNKSQSALGTAIERLSSGLRINSAKDDAAGQAIANRFTANIKGLTQASRNANDGISIAQTTEGALNEINNNLQRVRELAVQSANSTNSQSDLDSIQAEITQRLNEIDRVSGQTQFNGVKVLAQDNTGGSAPPHALSGGSGTMNFVARRGGSAKFVAAWTLKAAAGGSHLQGFVNLKGGSAKFVAAWTLKAAAGGSALYRRLTLIGGSAKFVAAWTLKAAAGGSYIRAKWINWGGSYQHLPPFRFGGSLGNTVNNLTSARSRIEDSDYATEVSNMSRAQILQQAGTSVLAQANQVPQNVLSLLR

**Table 5:** Physiochemical Properties of the full-fledged vaccine

Physicochemical characteristics	Values/description
Number of amino acids	320
Molecular weight	33,544.51
Instability index	37.63 (stable)
Aliphatic index	86.16
Theoretical pI	10.41
Extinction coefficient	33460
Total number of negatively charged residues (Asp + Glu)	17
Total number of positively charged residues (Arg + Lys)	30
Estimated half-life	30 h in mammalian reticulocytes, >20 h in yeast, and > 10 h in *E. coli*
Total number of atoms	4712
Grand average of hydropathicity (GRAVY)	− 0.225
Solubility determined using solpro server	Soluble (0.510037 probability)
Allergenicity	Non-allergen
Antigenicity using VaxiJen sever	Antigen (VaxiJen score 0.4892)

### Predicted secondary and tertiary structure

**Fig. 3:**
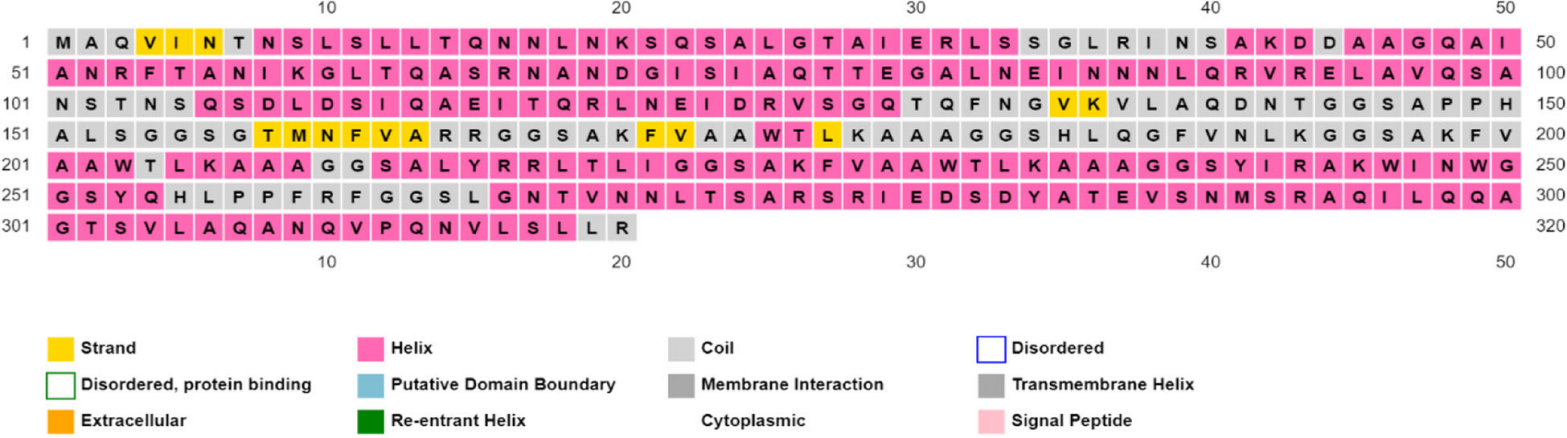
Full-fledged vaccine sequence and secondary structure description

**Fig. 4:**
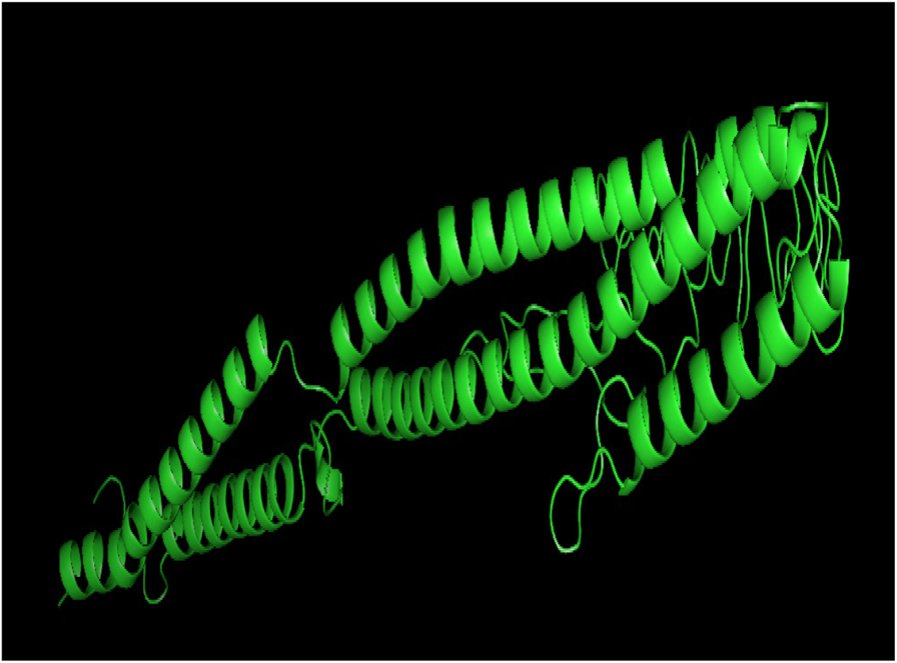
The 3D structure of vaccine construct

**Fig. 5:**
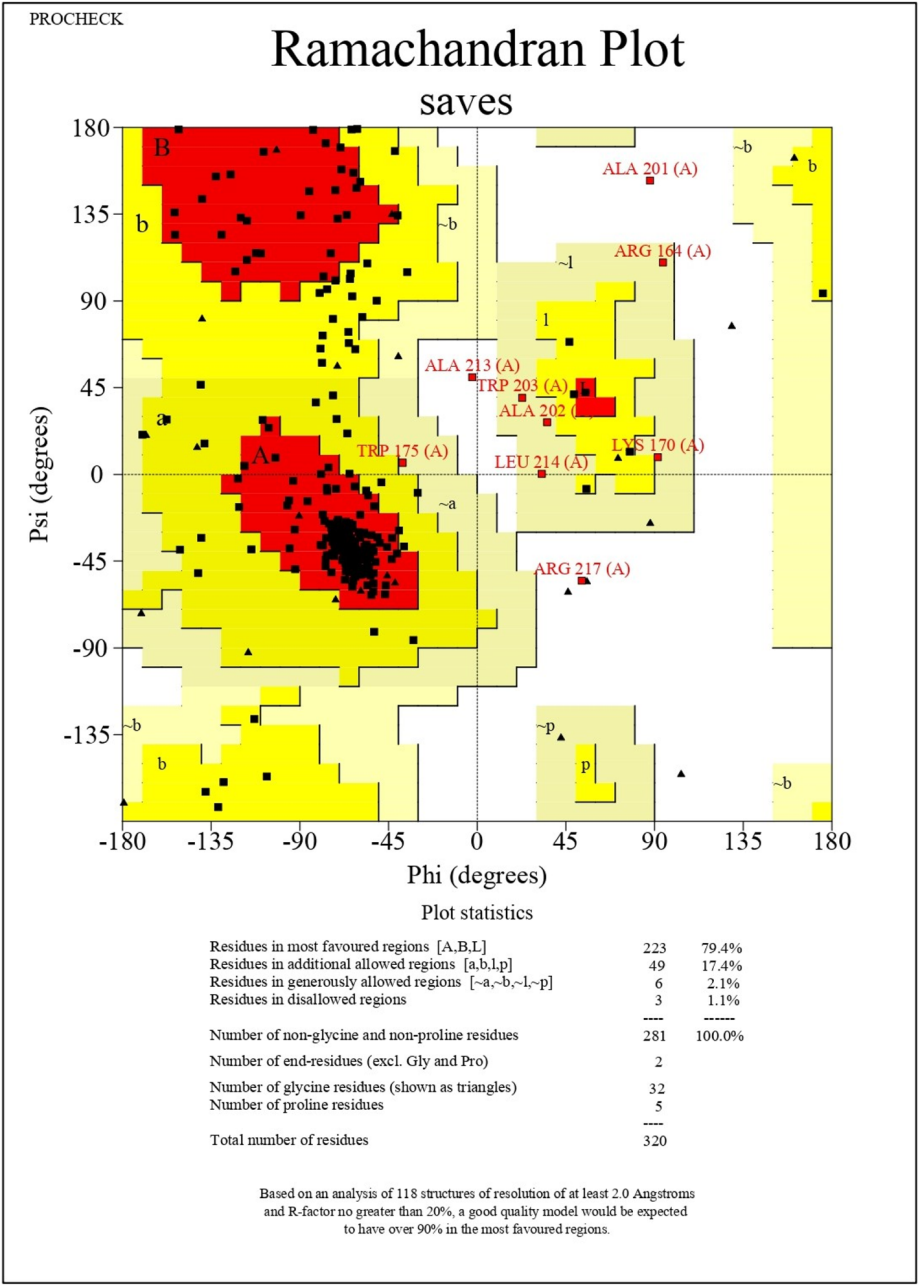
Ramachandran plot for a full-fledged vaccine against canine circovirus

The PSIPRED computed secondary structure of the vaccine contains beta-sheets, alpha helices, and coils are visualized in Fig. 3. The I-TASSER predicted best tertiary structure of the vaccine model had a *C*-score of − 0.60 and RMSD is 7.7 ± 4.3A. *C*-score should be in the range of − 5 to 2. The 3D structure of the vaccine construct is shown in Fig. 4. The MolProbity and Procheck validated the quality of the tertiary structures of vaccine; Ramachandran plot is shown in Fig. 5. Overall, 98.9% of all the residues were in allowed or favored region. Out of 320 residues, 281 residues were non-glycine and non-proline.

### Codon adaptation and vaccine in silico cloning

**Fig. 6:**
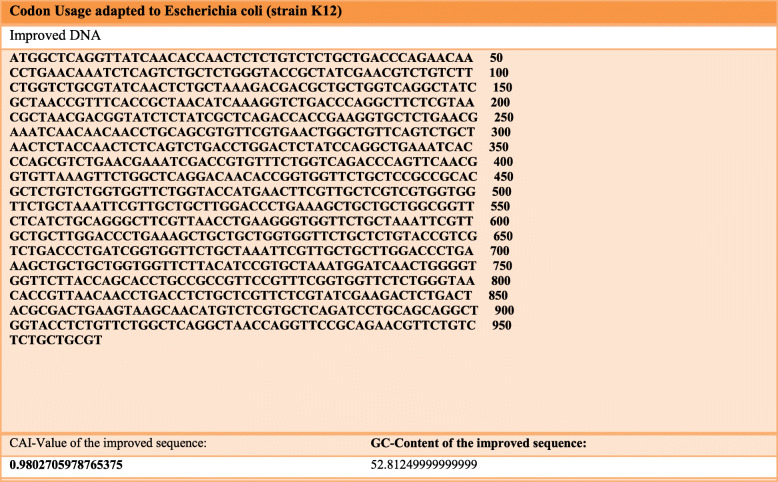
Codon adaptation index for the gene of a finalized full-fledged vaccine construct against canine circovirus

For efficient cloning and expression of the vaccine in expression vectors, codon optimization is imperative. The codon-optimized JCAT vaccine construct had a CAI value of 0.98 and a GC content of 52.81. In Fig. 6, CAI and genetic inspectional set for a full-fledged vaccine is provided.

**Fig. 7:**
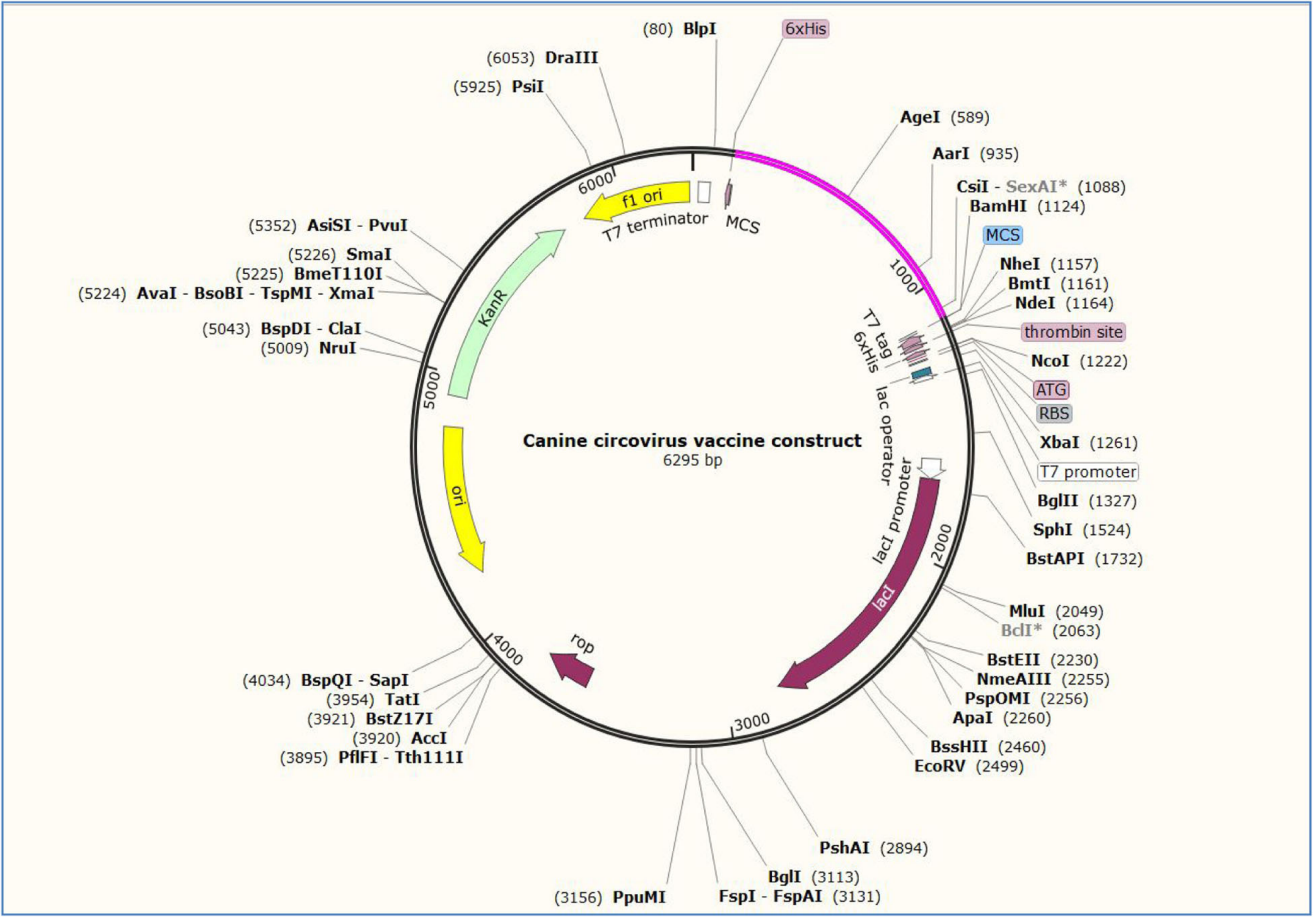
Vaccine constructs in pET28a (+) vector: SnapGene restriction cloning module developed by Insightful Science was used for in silico cloning. During cloning, the codon-optimized vaccine sequence was inserted between XhoI (158) and EcoRI (192) restriction sites of the pET28a (+) vector (vaccine construct is shown in magenta color)

During cloning, the codon-optimized vaccine sequence was inserted between XhoI (158) and EcoRI (192) restriction sites of pET28a (+) vector, as represented in Fig. 7.

## Discussion

There are two key inversely organized ORFs for *Circoviruses* that encode the replicase protein and capsid protein respectively [7, 10]. The replication-associated protein (Rep), which has sequence motif characteristic of proteins involved in rolling circle replication (RCR) [15], is the most conserved circovirus protein and is involved in the replication of the virus. In SARS-CoV-2, it has been reported that the replicase protein plays an important role in viral pathogenesis apart from its importance in viral replication process [14]. As replicase protein is highly conserved and plays an important role in pathogenesis, it can be a good candidate to predict epitopes for designing a vaccine construct. The capsid-associated protein (Cap) is the structural component of the viral capsid and is the main antigenic protein of canine circoviruses because it uses repeated subunits to compose the entire capsid structure of the virus; Cap is significantly divergent and is characterized by an N-terminal region rich in basic amino acids that may provide DNA binding activity [40]. Previously, three epitopes, NKPWH, QSLFFF, and KHSRYFT, were predicted from the capsid protein of Porcine circovirus type 3 (PCV-3) [41]. These epitopes were highly conserved B-cell epitopes and could be used to develop vaccine against PCV-3 [41]. In another study the capsid protein has been targeted to develop vaccine against dengue-2 virus [13]. This capsid-based vaccine developed cell-mediated immunity in monkeys [13]. In this study, these two proteins of canine *circovirus*, replicase and capsid proteins, were targeted to design a multivalent epitope-based vaccine by immunoinformatic approach. The immunoinformatic approach has been previously used to design vaccine candidates against other animal, fish, and poultry pathogens such as *Avian avulavirus*, foot and mouth disease virus, seven banded grouper nervous necrosis virus, and *Mycoplasma gallisepticum* [42–45]. However, there has been no report of development of vaccine candidate against canine circovirus by targeting its capsid and replicase protein by immunoinformatic approach so far. The in silico immunoinformatic approach has become the first step in developing vaccine candidates because of its cost-effective and time-saving manner [16, 46, 47]. This approach can also help to overcome limitations such as genetic variations, antigenic shifts, and antigenic drifts during the vaccine development process [48].

ViPR database was used for gathering information regarding viral proteome. Specific viral replicase and capsid proteins FASTA sequences retrieved from the NCBI database. The retrieved protein sequence data was analyzed by the NetMHCpan-4.1 webserver to predict epitopes that could bind with DLA molecules. The database also includes information about antigens of dog leukocytes (dog leukocyte antigen (DLA)) [27]. To predict the epitopes, dog leukocyte antigens DLA-8803401, DLA-8850101, and DLA-8850801 were selected because they are part of the major histocompatibility complex in dogs and are associated with the regulation of antigens in the immune system. Then, the epitopes which could bind strongly with DLA molecules were evaluated for their antigenic potential by VaxiJen webserver. The epitopes which had VaxiJen score above 0.7 were selected for further analysis as they are considered to be highly antigenic [49]. Altogether 5 epitopes were identified as highly antigenic: YQHLPPFRF, YIRAKWINW, ALYRRLTLI, HLQGFVNLK, and GTMNFVARR. Then, the structure of these antigenic epitopes was predicted. The interaction of these epitopes with the DLA alleles was evaluated by molecular docking study. The negative binding energy between the interactions of epitopes with the DLA molecules implies stable interactions and good binding affinity [16]. Furthermore, the molecular dynamics simulation analysis also corroborated that the interactions among the epitopes and the DLA alleles were stable. After the selection of the epitopes, they were linked by GGS linkers to adjuvants such as RS09 (APPHALS) and N and C terminal sequence of *Salmonella dublin* flagellin protein along with PADRE sequence to design the final vaccine construct. These linker and adjuvants have been previously used in multivalent epitope-based vaccine design of herpes simplex virus and human papillomavirus, *Candida auris*, human cytomegalovirus, and dengue virus [16, 17, 22, 23, 26, 50]. PADRE (AKFVAAWTLKAAA) sequence was added to enhance the stability and potency of the vaccine construct [50, 51]. The physiochemical properties like stability, number of amino acids, molecular weight, isoelectric point, aliphatic index, and number of positively and negatively charged amino acids and various other properties of the vaccine were also determined by the ExPASy ProtParam web server [34]. The final vaccine construct has 320 amino acids and it was predicted to be stable, soluble, antigenic, and non-allergic by different webservers. To determine the secondary structure of the vaccine, PSIPRED web server was used [36]. This web server predicted the presence of coils, strands, and helix in the final canine circovirus vaccine construct. The tertiary structure of the vaccine was determined by using the I-TASSER server. The analysis of the tertiary structure by Ramachandran plot showed that that quality of the 3D model of vaccine construct was good. Most of the residues (90.9%) were in allowed or favored region. The quality of the vaccine construct was also analyzed by *C*-score. Usually the *C*-score should be between the range of −5 and 2 [37]. Higher *C*-score implies good quality of the tertiary structure of the protein [37]. Here, the *C*-score of the vaccine construct was predicted as −0.60 which implies that the protein tertiary structure was of good quality. Finally, the in silico cloning analysis showed that the vaccine can be cloned and expressed in *Escherichia coli* for commercial large production. The results obtained in this study show that the vaccine construct could be a good candidate in protecting dogs from canine circovirus by generating robust immune response. However, these claims need further validation by performing different in vitro and in vivo experiments.

## Conclusions

In this research, we designed a multi epitope-based vaccine against canine circovirus by deploying immunoinformatic approach. We determined 5 epitopes YQHLPPFRF, YIRAKWINW, ALYRRLTLI, HLQGFVNLK, and GTMNFVARR from the capsid and replicase proteins of canine circovirus that were successfully used to craft a full-fledged vaccine construct using various in silico tools. This help in reducing time taking trial and error peptide screening for identifying vaccine candidates. This is very cost-effective method and easy to conduct before wet-lab trials. Such methods open new dimensions in veterinary research.

## Data Availability

All data is provided in the manuscript.

## Abbreviations

CaCV: Canine circovirus
RMSD: Root mean square deviation
RMSF: Root mean square fluctuation
ACE value: Atomic contact energy value
MD: Molecular dynamics
Rep: Replication-associated protein
Cap: Capsid-associated protein
SARS-CoV-2: Severe acute respiratory syndrome-coronavirus 2
